# Primary care physician perspectives on barriers to diagnosing axial Spondyloarthritis: a qualitative study

**DOI:** 10.1186/s12875-020-01274-y

**Published:** 2020-09-29

**Authors:** Kate L. Lapane, Sara Khan, Divya Shridharmurthy, Ariel Beccia, Catherine Dubé, Esther Yi, Jonathan Kay, Shao-Hsien Liu

**Affiliations:** 1 Division of Epidemiology, Department of Population and Quantitative Health Sciences, University of Massachusetts Medical School, 368 Plantation Street, Worcester, MA 01605 USA; 2 Sherman Center, University of Massachusetts Medical School, 55 Lake Avenue North, 6th floor, Worcester, MA 01655 USA; 3 Clinical and Population Health Research Program, Graduate School of Biomedical Sciences, University of Massachusetts Medical School, 368 Plantation Street, Worcester, MA 01605 USA; 4 Novartis Pharmaceuticals Corporation, 59 Route 10, East Hanover, NJ 07936 USA; 5 Division of Rheumatology, Department of Medicine, University of Massachusetts Medical School, Worcester, 55 Lake Avenue North, Worcester, MA 01605 USA; 6 Division of Rheumatology, UMass Memorial Medical Center, 119 Belmont St, Worcester, MA 01605 USA

**Keywords:** Back pain, Diagnosis, Primary care, Qualitative research

## Abstract

**Background:**

The average delay in diagnosis for patients with axial spondyloarthritis (axSpA) is 7 to 10 years. Factors that contribute to this delay are multifactorial and include the lack of diagnostic criteria (although classification criteria exist) for axSpA and the difficulty in distinguishing inflammatory back pain, a key symptom of axSpA, from other highly prevalent forms of low back pain. We sought to describe reasons for diagnostic delay for axSpA provided by primary care physicians.

**Methods:**

We conducted a qualitative research study which included 18 US primary care physicians, balanced by gender. Physicians provided informed consent to participate in an in-depth interview (< 60 min), conducted in person (*n* = 3) or over the phone (*n* = 15), in 2019. The analysis focuses on thoughts about factors contributing to diagnostic delay in axSpA.

**Results:**

Physicians noted that the disease characteristics contributing to diagnostic delay include: back pain is common and axSpA is less prevalent, slow progression of axSpA, intermittent nature of axSpA pain, and in the absence of abnormal radiographs of the spine or sacroiliac joints, there is no definitive test for axSpA. Patient characteristics believed to contribute to diagnostic delay included having multiple conditions in need of attention, infrequent interactions with the health care system, and “doctor shopping.” Doctors noted that patients wait until the last moments of the clinical encounter to discuss back pain. Problematic physician characteristics included lack of rapport with patients, lack of setting appropriate expectations, and attribution of back pain to other factors. Structural/system issues included short appointments, lack of continuity of care, insufficient insurance coverage for tests, lack of back pain clinics, and a shortage of rheumatologists.

**Conclusion:**

Primary care physicians agreed that lengthy axSpA diagnosis delays are challenging to address owing to the multifactorial causes (e.g., disease characteristics, patient characteristics, lack of definitive tests, system factors).

## Background

The average delay in diagnosis for patients with axial spondylarthritis (axSpA) [1–3] ranges from 7 to 10 years [4–8], with estimates as high as 13 years between symptom onset and diagnosis in the United States [9]. The significant diagnostic delay is a well-known feature of spondyloarthritis management [10]. Patients with longer delay in diagnosis (e.g., > 5 years) display more structural damages and limited spinal mobility [11]. In addition, patients may experience distress, depression, and desperation associated with their prolonged search for diagnosis and treatment [12]. Therefore, the improvement of delay in diagnosis for axSpA may slow disease progression and thus avoid or delay serious disability due to the early treatment interventions and access to care [13].

The delay in diagnosis for axSpA is multifactorial including characteristics of the patients, providers, healthcare system, and disease itself. For instance, patients may seek care from different medical professionals without guidance due to varied disease manifestations of axSpA or the lack of proper access to care (e.g., limited rheumatologists). Despite the use of sensitive imaging tool such as magnetic resonance imaging, the delayed diagnosis for patients with non-radiographic axSpA is still significant [14]. On the other hand, physicians may also play an important role in the early diagnosis of axSpA. Despite the low awareness of axial SpA among non-rheumatologist physicians [13], most axSpA patients are diagnosed by non-rheumatologists [15], with rheumatologists having diagnosed only 37% between 2000 and 2012 [16]. Primary care physicians may have difficulty discriminating inflammatory back pain from other types of back pain and may be unaware of other features that are important in making a diagnosis of axSpA.

In this qualitative research study, we sought the perspectives of primary care physicians to gain a better understanding of the reasons for diagnostic delay in primary care settings. We chose a qualitative approach because qualitative data frequently yield surprising insights and provide in-depth detail about participants’ perceptions and experiences that quantitative data often cannot [17]. Such foundational knowledge may be useful in developing interventions to reduce the delay in diagnosis of axSpA.

## Methods

The University of Massachusetts Medical School Institutional Review Board approved this study.

### Study design

The physician in-depth interviews were part of a larger qualitative study- The **Sp**ondylo**A**rthritis **S**creening and **E**arly **D**etection (SpA-SED) Study. Our protocol was guided by best practices for the conduct and reporting of qualitative research (COREQ) [18]. The appropriately completed COREQ checklist for the current study is included (Appendix A).

### Participant recruitment

We recruited 18 primary care physicians (a sample size likely adequate to achieve saturation) who were willing to participate in a recorded, 60-min in-depth discussion. We used purposive sampling to enroll equal numbers of men and women and family medicine and internal medicine physicians. Thirty-four participants including those who were known to the research team or their colleagues (approached by emails) or who were identified through state and regional primary care professional societies (face-to-face) were invited to participate. Six declined, nine were non-responders, and one physician was not selected because of our need for balance by gender and type of physician. All 18 participants participated in scheduled interviews and completed the study. Participants were offered a cash card of $300 to compensate for their time.

### Study setting

The interviews were conducted either in person in a private conference room in Rhode Island or Massachusetts (*n* = 3) or over the phone (*n* = 15) between February–May 2019. We conducted phone interviews using Zoom.

### Interview guide

A multidisciplinary team developed the interview guide (< 60 min interviews, Appendix B). Questions included experience with back pain, how back pain is evaluated, what laboratory tests are ordered when axSpA is suspected, what referrals participants would make, awareness of axSpA, speculation about what contributes to diagnostic delay in axSpA, etc. The average length of the interviews was 47.1 min (standard deviation: 11.3).

### Conduct of interviews

Interviews were conducted by experienced, trained personnel (KLL, DS), with an observer from the research team participating and taking notes. For one interview, the research interviewer (KLL) had previously worked as an investigator on a research project with the physician participant (> 5 years previously). Research staff were trained to assure standardized data collection. The protocol was pilot tested with one physician. The researchers conducting the interviews reported no obvious bias but did report prior assumptions that lack of awareness of axSpA and lack of time would emerge as leading barriers to axSpA screening in primary care settings. No repeat interviews were conducted.

### Data collection and management

The study data were collected and managed using REDCap electronic data capture tools hosted at the University of Massachusetts Medical School [19]. REDCap (Research Electronic Data Capture) is a secure, web-based application designed to support data capture for research studies, providing: 1) an intuitive interface for validated data entry; 2) audit trails for tracking data manipulation and export procedures; 3) automated export procedures for seamless data downloads to common statistical packages; and 4) procedures for importing data from external sources.

### Analytic strategy

We conducted a group method of data analysis known as immersion / crystallization [20]. Four team members independently listened to selected interview recordings, read in-depth transcripts, and wrote analytic notes for each. The transcript texts were subjected to line-by-line coding with NVivo qualitative software [21]. The codebook was modified by team consensus. Searches for alternative interpretations were conducted again and discussed before final decisions were made about how to report the findings of the study. This manuscript focused on one parent node in our coding scheme: Reasons for Diagnostic delay.

### Feedback from participants

We prepared a preliminary report of our findings and emailed participants who indicated they were willing to review and provide feedback (Appendix C).

## Results

**Fig. 1:**
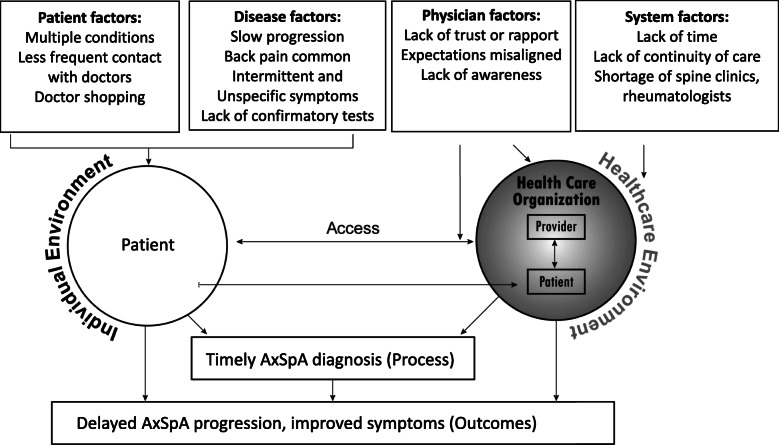
Factors influencing timely diagnosis of axSpA

Of the participants, 44% were women, 66.7% were non-Hispanic white and 83.3% attended an allopathic medical school in the United States (Table 1). All physicians considered that the length of diagnostic delay was problematic, and all agreed that this delay was unacceptable. Physicians believed that multiple factors were contributing to the diagnostic delay in axSpA. Figure 1 depicts the multifactorial components perceived by primary care providers participating in the study. Barriers to early diagnosis included disease characteristics (e.g., slow disease progression; Table 2), patient factors (e.g., multiple other conditions, back pain not chief complaint, Table 3), physician characteristics (e.g., lack of rapport/trust, Table 4), and structural/system issues (e.g., lack of time, Table 5). Primary care physicians noted that, because back pain is multifactorial and very common and axSpA is not, axSpA may be missed. Physicians explained that the slow progression of the disease may contribute to the diagnostic delay. The intermittent nature of symptoms and range of symptoms severity related to axSpA also was noted as a factor contributing to diagnostic delay. Additionally, physicians pointed out that it takes time for radiographic evidence to appear and that there are no other definitive diagnostic tests.

**Table 1:** Characteristics of physicians

	Total (*n* = 18)
Age (years), mean (SD)	46.8 (12.6)
Women, %	44.4
Race/ethnicity, %	
Non-Hispanic, White	66.7
Non-Hispanic, Black	5.6
Hispanic	5.6
Other	22.2
Trained at, %	
US Allopathic school	83.3
US Osteopathic school	0
Foreign medical school	16.7
Years in practice, mean (SD)	15.9 (13.0)
Practice characteristics: % (check all that apply)	
Individual	0
≤ 5 physicians	27.8
≥ 6 physicians	55.6
Hospital-based practice	38.9
Academic affiliation	66.7
Confidence in distinguishing inflammatory versus mechanical back pain, %	
Not confident	16.7
Somewhat confident	38.9
Very confident	33.3
Extremely confident	11.1
Knowledge of inflammatory back pain classification criteria, %	
Calin criteria	0
ASAS criteria	16.7
Berlin criteria	0

*ASAS* Assessment of Spondyloarthritis International Society

*SD* Standard Deviation; Percentages may exceed 100% due to rounding

**Table 2:** Disease characteristics contributing to diagnostic delay for patients with axSpA

	Sample Quotes
Back pain is very common, axSpA is not	D25: in primary care general practice, … there’s the old adage when you hear hoofbeats, think horses, not zebras. So, ankylosing spondylitis is a zebra. D10: it’s certainly not on the top three things I think of when somebody comes in and says my back hurts. So, and, you know, I’m not sure if I’m just missing it because I’m not looking for it or is it just relatively rare
Slow disease progression	D34: Or things behave similarly early on, and advanced imaging or blood tests may be easy not to do based on let’s try this, let’s try that, and if things don’t get better, that usually is that patients come back and other symptoms start arising. So it might take a while for more systemics to arise that might put that on the radar of the physician to work up further. D5: You know, it progresses over time and you -- before you get to bamboo spine.
Intermittent nature of pain with axSpA	D24: Well, patients are often, when they’re uncomfortable -- well, unless they’re getting, you know, they’re having a flare and then nothing for a few years and then getting a flare, nothing. D22: a lot of pain generally improves with time, especially acute -- either acute pain or acute flareup sorts of pain, so it kind of gives the patient, you know, a couple of weeks. Usually by that time the pain always -- almost always goes away.
Lack of characteristic radiographic appearances	D11: Part of the delay, one would think, is probably related to the lack of characteristic radiographic appearance of -- sacroiliitis sounds great for you and I to talk about but the radiograph isn’t always, you know, knock your socks off. It takes a while until you start obscuring the joint margin. D22: The patient was there for something else and he said that okay, I’ve been having a lot of stiffness in the morning and that was – he had typical symptoms but when we did his x-ray there was nothing specific.
No definitive test for diagnosis	D24: I would probably move to imaging before I would get something like a B27 simply because -- and, I’ll be honest, I don’t know the percentages here, … we have more back pain from arthritis and disks and it’s such a common problem. The amount of people who are going to have a positive B27 is fairly low. D3: I have sent them for a work up. Most of the time it’s been negative.

**Table 3:** Patient characteristics contributing to diagnostic delay for patients with axSpA

	Sample Quotes
Patients may not interact frequently enough with the health care system	D1: And it’s also possible that it might occur in people with less access to healthcare because of socioeconomic reasons. D23: It could be that patients aren’t going in until a lot later because they don’t want to see a doctor, they’re nervous about the diagnosis …
Patients having multiple issues to discuss	D7: They bring in a list and then they say okay, I have a list and if I have a list you have to pay attention to it all and then I’m like when we’re at number ten or number 13, we’re not going there. But we realize it’s a negotiation and the biggest challenge is when you just don’t have time. D27: I mean, if patients haven’t accessed the medical system often enough or at all, they will come in with a -- like a laundry list of six or seven or eight problems, and I -- my approach is often, like, let’s talk about your top two.
Patients often add back pain onto the list at the last moment	D12: They’re clearly the visit’s scheduled for the back pain, and that’s the main theme of the discussion for that visit. … [it] also sometimes comes up when they’re here for something else and they would say, “Oh, by the way, my back has been hurting,” or, “I have a bad back”.
Patients doctor shop and concern about malingering	D22: A lot of patients do go from one doctor to the other and that always doesn’t help because the next doctor, they’ve seen the patient for 1 month only and then they start the whole process again, and the patient goes through a battery of tests. D23: well, but they also hop physicians because they feel like what they’re doing isn’t helping me, but I think that’s the problem is they’re assuming that there aren’t other things that can be addressed or looked at.

**Table 4:** Physician characteristics that may reduce diagnostic delay for patients with axSpA

	Sample Quotes
Establish trust and good rapport with patients	D23: I think also if a patient actually establishes rapport with the doctor and there’s a good doctor-patient relationship and they are familiar with each other, if something like this does crop up, it is out of the ordinary based on what the doctor knows of the patient. D23: I think what it really comes down to just trust and open relationship with the doctor and the patient. D31: Some of my colleagues don’t listen well enough to patients and they miss things. Actively listening to the patient is going to be the most important thing I think any physician -- most important skill that I think any physician can possess, actively listening. Talking less and listening more. Because I always learned when I was a medical student that, you know, your patient is going to give you the diagnosis, all you have to do is listen.
Setting appropriate expectations	D3: But if its somebody who is coming in who has had chronic back pain, has had a lot of work up, has had good intervention, then I think that’s somebody where I’m starting to turn on my radar and thinking, “Okay, is there something else that I’m missing here that’s not just your run in the mill. D27: I would be seeing them in follow up and noticing that they didn’t resolve typically, and I would probably be -- in my next exam be doing a review of systems again to find those other systems that were involved.
Some physicians attribute back pain to other factors	D10: It’s certainly not on the top three things I think of when somebody comes in and says my back hurts. … I’m not sure if I’m just missing it because I’m not looking for it or is it just relatively rare. D24: they’re saying well, yeah, but I was in a car accident when I was 18 and I rolled the car and, you know, so my back has been hurting me off and on since I was then -- you know, now that I’m, you know, 35 and I’ve put on some weight and I don’t exercise anymore, my back is hurting.

**Table 5:** System/structural characteristics contributing to diagnostic delay for patients with axSpA

	Sample Quotes
Length of time for appointments needs to be longer	D27: Time is a constraint. I would love to have 30 min for every problem a patient comes in and then I think this wouldn’t be an issue.
Lack of continuity of care	D3: Well, I would actually wonder how many of those people were not getting regular care like with the same physician, like continuous care. So, you know a lot of say younger people who go to an urgent care center are like you know … “Oh, I’m here and I’ve got low back pain” and for the doctor it’s like, let me give them a shot of Toradol and send them on their way, “Here’s some anti-inflammatory meds, follow up with your primary care.” D32: They [patients] get kind of used to the pain, and they’ve kind of seen doctors who have examined them and said, hey, everything looks fine. You don’t need to keep coming in.
Costs of tests expensive	D24: I see so many people with back pain, a subset of those I test for autoimmune diseases, but even when I’m doing that, if I’m going to test or something like B27, I can’t really use it as a screening test because insurance doesn’t pay for those sort of tests just for a diagnosis of back pain … Uveitis, I think it will get covered, but, I mean, people get upset when they get a big bill; why did you do all these blood tests on me? D12: Once I see, yes, it’s heading in that direction, I’ll probably do blood work and their clear blood work, ANA, HLA-B27, which I kind of wait, because it might be an expensive.
Lack of back pain clinics	D11: Chronic back pain, no one wants to see them, no one wants to own them. D29: Yeah, so our orthopedists don’t see back, so only our neurologist will see our back patients, if we have anybody. I don’t think rheum sees back. D28: Oh, yeah; it’s the most difficult thing to -- the single worst referral is the referral for the rheumatologist.
Shortage of available rheumatologists	D31: The issue is we don’t have many rheumatologists at all. There’s a shortage. … the nearest rheumatologist, I think, is probably going to be at least an hour drive away or maybe like 45–50 miles. D24: We don’t have rheumatology in our county, so that’s an out of county referral and much harder to get an appointment.

A common theme was that patients do not interact frequently enough with the health care system and the lack of continuity of care (Table 3). Primary care physicians uniformly noted that patients often have multiple issues to discuss and back pain is mentioned only at the very end of the visit. Physicians did not discuss why patients waited until the last moment to share their complaints of back pain. Some physicians explained that they are likely to attribute back pain to other conditions (Table 4); most emphasized the need to establish trust and a good rapport with patients. The importance of setting appropriate expectations with patients was noted, since back pain may not be addressed adequately in one visit. Primary care physicians lamented that the limited amount of time available for appointments impedes their ability to diagnose axSpA in a timely fashion (Table 5). Further, physicians noted that the lack of continuity of care may contribute to diagnostic delay. Patient concerns about the expense of copayments expected when undergoing testing needed to evaluate axSpA may also contribute to the diagnostic delay. The lack of dedicated spine clinics and the shortage of rheumatologists were reported as barriers to timely diagnosis.

## Discussion

This study sheds light on the challenges faced by primary care physicians that may contribute to the delay in diagnosing axSpA, which typically presents with the insidious onset of back pain that often is attributed to other, more prevalent conditions [22, 23]. The subjective quality of pain may be difficult for patients to describe and is not easily measured objectively. In our study, primary care physicians noted that axSpA patients may not interact frequently enough with the health care system to facilitate a correct diagnosis. Consistent with previous research [16], lack of diagnostic criteria, lack of definitive biomarkers, and lack of radiographic confirmation of axSpA until later in the disease course [13, 24] were identified as factors contributing to axSpA diagnostic delay.

Several physician-related factors that may contribute to diagnostic delay were identified, including a lack of awareness about axSpA among non-rheumatology health care professionals [9]. Lack of trust, poor rapport, ineffective communication, and misaligned expectations between providers and patients were factors that likely contribute to diagnostic delay. Among patients with immune-mediated inflammatory diseases, distrust of health care providers has been associated with poor medication adherence [25] while, conversely, good patient-provider communication has been shown to improve health outcomes [26]. Our participants also reported that patients may not realize that axSpA often is not diagnosed during a single visit, but rather involves a journey that occurs over a period of time. Physicians mentioned that infrequent contact with the health care system and lack of continuity of care contributed to the diagnostic delay. To our knowledge, research has not been conducted on whether discordant expectations between patients being evaluated for axSpA and their primary care physicians contributes to diagnostic delay. Nevertheless, improved alignment of patient expectations to care processes may improve patient continuity of care.

Primary care physicians mentioned system-level factors that impede timely diagnosis of axSpA, including the short appointment length and difficulty arranging rheumatology referrals. The median length of a primary care visit was 15.7 min, during which a median of six topics were covered with ~ 5 min spent discussing the primary complaint and 1.1 min on each remaining topic [27]. A musculoskeletal or rheumatologic condition accounted for the chief complaint in only about 8.3% of primary care visits [28], which is consistent with reports by providers in our study that mention of back pain may be withheld by patients until the end of the clinical encounter. Patients with axSpA often have multiple conditions, such as hypertension and depression, which might supersede back pain as the main focus of a primary care visit [29]. Given the challenges of reduce time with patients and increased complexity of patient issues, automated tools may be useful to primary care physicians to identify uncommon diseases such as axSpA.

Delays in diagnosis of axSpA also have been attributed to late referral of patients with inflammatory back pain by general practitioners to rheumatologists [15, 16, 30, 31]. However, the average wait for a rheumatology appointment is 4 months [32]. Difficulty accessing rheumatology care is exacerbated by the shortage of and decline in the number of practicing rheumatologists [33]. By 2025, the demand for rheumatologists is projected to exceed supply and a shortage of 3845 rheumatologists is predicted [34, 35]. This shortage results in burdens for patients, including excessive travel time – which may exceed 90 min for some patients [36]. The American College of Rheumatology has proposed multiple strategies to address this workforce shortage, but the extent to which these will be successful remains uncertain.

Limitations must be considered. Most of the participants practiced in Massachusetts and Rhode Island and had academic affiliations. Systems factors may be specific to local context. Physicians with academic affiliations may be more aware of axSpA. Patient factors were reported by the physician participants and are derived from experiences with their own patients. It is possible that physicians only had experience with patients with ankylosing spondylitis. Misinterpretations of direct quotes may have occurred, although the analytic approach used reduced this likelihood. However, despite these potential limitations, our findings appear to be aligned with previous research [16].

## Conclusions

Aiming to provide a better understanding of the reasons for diagnostic delay in primary care settings, this study identified primary care physicians’ perceptions of several root causes of diagnostic delay in axSpA. These contributing factors of delay in diagnosis for axSpA were believed to be multifactorial and were attributed to characteristics of patients, providers, the healthcare system, and the disease itself. While in our study primary care physicians felt that the diagnostic delay reported in the literature is lengthy, it must be noted that Primary care physicians believed that strategies to address these barriers and reduce delay in diagnosis of axSpA are needed. Early diagnosis of axSpA is difficult and as such solutions to shorten the diagnostic journey must consider the multitude of contributing factors (i.e., patient, disease, physician, system).

## Data Availability

The data analyzed during the current study are not publicly available due to the nature of the qualitative data. We also did not include the sharing of data in our informed consent procedures, so we are unable to share our qualitative data.

## Appendix A

**Table 6:** COREQ (COnsolidated criteria for REporting Qualitative research) Checklist

Domain and Items	Page Number Reported
**Domain 1: Research team and reflexivity**	
**Personal characteristics**	
1. Interviewer/facilitator Which author/s conducted the interview or focus group?	Page 5. The first and third-named authors listed conducted the interviews.
2. Credentials What were the researcher’s credentials?	Page 1.
3. Occupation What was their occupation at the time of the study?	Page 1.
4. Gender Was the researcher male or female?	N/A.
5. Experience and training What experience or training did the researcher have?	Page 5.
**Relationship with participants**	
6. Relationship established Was a relationship established prior to study commencement?	This is acknowledged on Page 5: “Thirty-four participants including those who were known to the research team or their colleagues (approached by emails) or who were identified through state and regional primary care professional societies (face-to-face) were invited to participate.”
7. Participant knowledge of the interviewer What did the participants know about the researcher? e.g. personal goals, reasons for doing the research	Following on from above, some participants known to the research team were recruited. While a few of the participants knew the researcher (KLL), none knew of any personal goals or reasons for doing the research. Participants received a fact sheet about the research (including its aims and rationale) and had the opportunity to ask the researcher additional questions prior to deciding if they wished to participate. Page 11: “All participants provided informed consent.”
8. Interviewer characteristics What characteristics were reported about the interviewer/facilitator? e.g. Bias, assumptions, reasons and interests in the research topic	Page 5–6.
**Domain 2: study design**	
**Theoretical framework**	
9. Methodological orientation and Theory What methodological orientation was stated to underpin the study? e.g. grounded theory, discourse analysis, ethnography, phenomenology, content analysis	Page 6.
**Participant selection**	
10. Sampling How were participants selected? e.g. purposive, convenience, consecutive, snowball	Page 5.
11. Method of approach How were participants approached? e.g. face-to- face, telephone, mail, email	Page 5.
12. Sample size How many participants were in the study?	Page 5.
13. Non-participation How many people refused to participate or dropped out? Reasons?	Page 5.
**Setting**	
14. Setting of data collection Where was the data collected? e.g. home, clinic, workplace	Page 5.
15. Presence of non-participants Was anyone else present besides the participants and researchers?	Page 5. “Interviews were conducted by experienced, trained personnel (KLL, DS), with an observer from the research team participating and taking notes.”
16. Description of sample What are the important characteristics of the sample? e.g. demographic data, date	Page 5 & 7.
**Data collection**	
17. Interview guide Were questions, prompts, guides provided by the authors? Was it pilot tested?	Page 5–6.
18. Repeat interviews Were repeat interviews carried out? If yes, how many?	Page 6. “No repeat interviews were conducted.”
19. Audio/visual recording Did the research use audio or visual recording to collect the data?	Page 5. “We conducted phone interviews using Zoom.”
20. Field notes Were field notes made during and/or after the interview or focus group?	Page 5.
21. Duration What was the duration of the interviews or focus group?	Page 5.
22. Data saturation Was data saturation discussed?	Page 5.
23. Transcripts returned Were transcripts returned to participants for comment and/or correction?	N/A.
**Domain 3: analysis and findings**	
**Data analysis**	
24. Number of data coders How many data coders coded the data?	Page 6.
25. Description of the coding tree Did authors provide a description of the coding tree?	Page 6.
26. Derivation of themes Were themes identified in advance or derived from the data?	Page 6.
27. Software What software, if applicable, was used to manage the data?	Page 6.
28. Participant checking Did participants provide feedback on the findings?	Page 6.
**Reporting**	
29. Quotations presented Were participant quotations presented to illustrate the themes / findings? Was each quotation identified? e.g. participant number	Page 15–19: Table 2, 3, 4 and 5
30. Data and findings consistent Was there consistency between the data presented and the findings?	Page 7; Page 15–19: Tables 2, 3, 4 and 5
31. Clarity of major themes Were major themes clearly presented in the findings?	Page 15–19: Tables 2, 3, 4 and 5
32. Clarity of minor themes Is there a description of diverse cases or discussion of minor themes?	N/A.

## Appendix B

**Table 7:** Codebook for primary care physician interviews

Name	Description
Patient characteristics	Any mention of these sociodemographic factors, as they relate to diagnosing, “textbook cases”, thoughts about axSpA, etc. a. Age b. Weight-status/obesity c. Sex/gender d. Socioeconomic status e. Employment f. Multiple comorbidities
Approaches to diagnosing axSpA	All quotes falling under this code should refer to axSpA specifically, not general back pain, acute injuries, etc. a. Typical presentation of axSpA b. Clues within workup pointing towards axSpA c. Triggering events d. Medical history e. Imaging and lab tests if axSpA is suspected f. Ruling out other diagnoses
Reasons for diagnostic delay	a. Back pain is common and causes are multifactorial b. axSpA is rare and has vague/nebulous symptoms c. Challenges faced by PCPs regarding rare/specialized conditions d. Structural barriers within healthcare system e. Wary of drug seeking behavior
Overcoming delays	Factors that could reduce the diagnostic delay (note: can include the need to implement a screening process)
Referrals	a. Access b. Physical therapy c. Rheumatology d. Other specialists
Screening	a. Barriers = what makes screening difficult b. Facilitators = what would make screening easier or likely to be implemented c. General thoughts = current approaches, brainstorming other strategies currently being used or that could be used (e.g., NLP)
axSpA Awareness	Brief response to question about whether they and/or colleagues are aware of axSpA and whether they believe the diagnostic delay is an issue
Screening questions	a. Question 1: Have you suffered from back pain for more than 3 months? b. Question 2: Did your back pain start when you were aged 40 or under? c. Question 3: Did your back pain develop gradually? d. Question 4: Does your back pain improve with exercise? e. Question 5: Do you find there is no improvement in your back pain when you rest? f. Question 6: Do you suffer from back pain at night which improves upon getting up? g. Recommendations

## Appendix C

**Table 8:** Feedback from physician participants on preliminary report

Participant	Comments
D31	Thank you for sharing the preliminary results of the study on inflammatory spondyloarthropathies and screening mechanisms for primary care. I was pleased to have participated. I do have additional recommendations from a physician perspective but am delight to learn that the next stage of your analysis will move to getting the input of patients through real-time interviews. Congratulations on excellent work and bringing importance to an often overlooked arthropathy in our clinics due to misdiagnosis. We must be willing, as physicians, to take detailed medical histories and do physical examinations to get to root, and please do spread knowledge of the screening tool and considering incorporating into medical apps such as Medscape and QxMD. That will help.
D34	Overall the summary for each section the comments make sense with regard to: - the limitation of history taking questions to help dx axSpA - the difficulties in making the diagnosis of axSpA - the limitations of screening questions\screening tools axSpA is a difficult dx to make given how common back pain is seen in the primary care’s office. Even using screening tools either filled out by the patient or used by the provider still those would still have significant limitations making them not clinical useful in a busy out-pt. setting.

## Abbreviations

axSpa: axial spondyloarthritis
COREQ: Conduct and reporting of qualitative research
REDCap: Research Electronic Data Capture
SpA-SED: The **Sp**ondylo**A**rthritis **S**creening and **E**arly **D**etection
